# The influence of attention and reward on the learning of stimulus-response associations

**DOI:** 10.1038/s41598-017-08200-w

**Published:** 2017-08-22

**Authors:** Devavrat Vartak, Danique Jeurissen, Matthew W. Self, Pieter R. Roelfsema

**Affiliations:** 1 Department of Vision and Cognition, Netherlands Institute for Neuroscience, Royal Netherlands Academy of Arts and Sciences, Amsterdam, The Netherlands; 2 Department of Integrative Neurophysiology, Centre for Neurogenomics and Cognitive Research, VU University, Amsterdam, The Netherlands; 3 Psychiatry Department, Academic Medical Center, Amsterdam, The Netherlands; 4 Department of Neuroscience, Zuckerman Mind Brain Behavior Institute, Columbia University, New York, USA

## Abstract

We can learn new tasks by listening to a teacher, but we can also learn by trial-and-error. Here, we investigate the factors that determine how participants learn new stimulus-response mappings by trial-and-error. Does learning in human observers comply with reinforcement learning theories, which describe how subjects learn from rewards and punishments? If yes, what is the influence of selective attention in the learning process? We developed a novel redundant-relevant learning paradigm to examine the conjoint influence of attention and reward feedback. We found that subjects only learned stimulus-response mappings for attended shapes, even when unattended shapes were equally informative. Reward magnitude also influenced learning, an effect that was stronger for attended than for non-attended shapes and that carried over to a subsequent visual search task. Our results provide insights into how attention and reward jointly determine how we learn. They support the powerful learning rules that capitalize on the conjoint influence of these two factors on neuronal plasticity.

## Introduction

We can learn new tasks by listening to a teacher or by reading the instructions, but we also encounter many tasks that require us to learn by trial and error. The mechanisms that permit us to learn how to map stimuli onto arbitrary responses are only partially understood. Here, we wish to investigate the factors that determine trial-and-error learning in human subjects. Does learning in human observers comply with reinforcement learning theories, which describe how animals and artificial systems learn from trial and error to optimize their behavior? What is the influence of selective attention in the learning process?

The goal of reinforcement learning is to find the policy that will maximize the amount of reward that is obtained by an agent while performing a task^1^. The reward structure of a task is presumably also the most important determinant of human and animal learning^2^. Reinforcement learning theories propose that the changes in connectivity in the brain or in an artificial neural network should depend on the reward prediction error (RPE), which is the difference between the amount of reward that is expected for a particular action and the amount that is obtained. If the action yields more reward than expected, the RPE is positive and connections should change such that the probability of choosing this action will increase in the future. Vice versa, if the outcome is disappointing, the RPE is negative and the probability of selecting the same action should decrease. The relevance of reinforcement learning theory for animal learning was boosted enormously when it became evident that there are neurons in the brain that code for the RPE. Schultz and his co-workers^3^ reported that dopamine neurons in the ventral tegmental area and the substantia nigra code for positive RPEs, and more recent studies reported that other neuromodulatory systems implicated in learning, such as acetylcholine^4^ are also sensitive to RPEs. It is even possible to use fMRI to measure neuronal correlates of RPEs in cortical and subcortical regions in the human brain^5^.

Another factor that influences learning is selective attention^6, 7^. A number of previous studies have used the “redundant-relevant cue” paradigm^8^ to study the role of attention in learning. In this paradigm, subjects learn to map stimuli onto responses, while seeing multiple cues in every trial that are all associated with the correct response. The information is redundant because subjects can, in principle, select the correct response based on only one of these cues and they can afford to ignore the others. The main finding is that if selective attention is directed to one of these cues during learning, this cue is learned and the others are not^6, 8^. This result implies that selective attention gates learning. However, the finding that learning only occurs for attended information is not undisputed, because other studies have reported that subjects also learn about unattended stimuli^9^. Subjects can even learn about stimuli that do not reach awareness, in particular when they are close to the threshold for perception and paired with a reward^10^. Thus, the precise contributions of the RPE and selective attention to the learning process remain to be established.

Interestingly, recent modeling studies^11, 12^ suggest that powerful learning rules are possible in the nervous system if selective attention and RPEs jointly determine synaptic plasticity^13^. These studies proposed that when subjects select a particular response, the brain areas involved in response selection feed a selective attention signal back to earlier processing levels, which “tags” the synapses that are involved in the stimulus-response mapping for plasticity. Support for an influence of response selection on sensory representations comes from eye movement research, where every eye movement causes a shift of attention to the stimulus that is the target for the eye movement^14^, and also from neurophysiological studies demonstrating that selection of a motor response in frontal cortex enhances the representations in visual cortex that gave rise to this response^15^. The hypothesis is that this attentional feedback signal renders a selective subset of synapses sensitive to the neuromodulatory systems that code for the RPE^11, 12^. The tagged synapses were involved in the stimulus-response mapping and they would then increase or decrease in strength depending on the sign and size of the RPE, whereas the synapses that did not receive this attentional feedback signal would remain untagged and their strength would remain the same. Interestingly, the conjoint influence of selective attention and the RPE gives rise to learning rules that are equivalent to the error-backpropagation rule used in deep learning^16^, which is of interest because error-backpropagation was previously thought to be biologically implausible. The computational power of these new biologically plausible learning rules permits the training of neural networks for many complex cognitive tasks^12^.

In the present study, we wished to address three questions: (1) Does reward influence learning when subjects learn to map new stimuli onto responses? (2) What is the influence of attention on learning? (3) Are there long term influences of the learning that carry over to later tasks? (4) Are the effects of reward magnitude on learning stronger for attended stimuli than for non-attended ones, as is predicted by the biologically plausible learning rules?

To address these questions, we designed three new tasks, which were based on the redundant-relevant cue paradigm^8^. All subjects participated in all three tasks. The first task required the subjects to map unfamiliar icons onto a motor response while they attended to some icons and ignored others. We orthogonally varied the reward that was associated with the different icons. The second task probed the stimulus-response associations that had formed during learning and the third task examined implicit effects of attention and reward on learning by using the icons as targets during a visual search paradigm. We found that subjects only learned about stimuli that are attended and that the precise amount of reward obtained upon correct performance had only a weak effect on the speed of learning. Reward and attention jointly determined the subjects’ efficiency in the later search task. Our results thereby provide new insights into how attention and reward jointly determine the formation of new stimulus-response associations.

## Results

Subjects (n = 16) performed three tasks in succession. Task 1 was an icon learning task where subjects learned new stimulus-response associations while we varied the subjects’ attention and the amount of reward given after a correct response. Task 2 was a probe task where we examined how well subjects had learned these new stimulus-response associations and task 3 investigated how attention and reward during task 1 influenced the efficiency of visual search for the now familiar icons.

### Icon learning task

**Figure 1:**
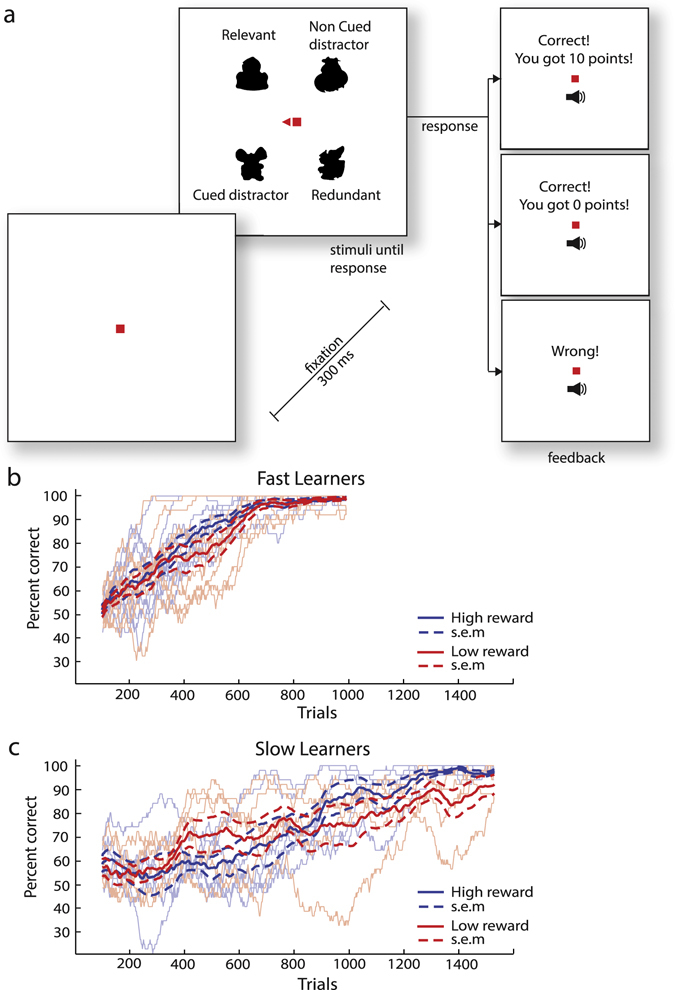
Shape learning task. (**a**) Trial structure. In every trial the participants saw four icons. They were instructed to pay attention to the side of the screen that was cued with a red triangle. Their task was to determine which of the two icons on the cued side was associated with a particular response button, by trial and error. They received auditory and visual feedback whether their response was erroneous or correct and on correct trials they also received feedback about the number of points that they gained (0 or 10 points). (**b**) Accuracy of the subjects that learned fast (within 1024 trials). Blue curves represent the accuracy for the high-reward icons and red curves the accuracy for the low-reward icons. The lighter curves show the accuracies of individual subjects (in a window of 50 trials) and the darker curves the average across subjects (N=10 fast learners). Dashed lines represent s.e.m. (**c**) Accuracy of the 6 subjects that learned more slowly and received additional training.

Subjects initially learned to associate icons with one of two response buttons (Fig. 1a). On every trial, the subjects saw a stimulus with four icons, two in the left hemi-field and two in the right hemi-field. During a block of 128 trials, a central triangle cued the subject to stably attend to the left or right hemi-field for the duration of the block. It was the subjects’ task to learn which of the two stimuli on the attended side was a target icon (relevant icon) and how it mapped onto one of two response buttons, while disregarding the other shape, which will be called “cued distractor” and was uninformative about the correct response. The information in the other, non-cued hemi-field was equally informative. On this side, we presented a redundant icon, which was coupled to the same response button as the target icon and a non-cued distractor that was not. Furthermore, for half of the icons, a correct response was followed by a high reward (10 points) with visual feedback and an accompanying sound. For the other icons, a correct response was followed by low reward (zero points), visual feedback and a sound indicating that the selected response button was correct. Incorrect responses were followed by visual feedback and a buzzer-sound (Fig. 1a). When a new block of 128 trials started, the triangle cue instructed subjects to attend the other side. All icons that were on the left when the cue pointed to the left were presented on the right when the cue pointed to the right, and vice versa for the icons in the other hemifield. Thus, half of the icons always appeared in the attended hemifield and the others in the unattended hemifield.

We stopped the learning task after 1024 trials if the subjects’ average accuracy in the last 256 trials was above 85%, a stopping criterion that was met by 10 out of 16 subjects (Fig. 1b). The other 6 subjects carried out an additional 512 trials and they then also reached an accuracy higher than 85% in the last 256 trials (Fig. 1c). Thus, all 16 subjects learned the task, and their average accuracy was 96.8% (s.e.m. 0.9%) in the last 256 trials. We next examined whether the reward level influenced the speed of learning. Figure 1b,c compares the learning curves for icons that were associated with a high and low reward, smoothed by computing accuracy in a moving window of 50 trials (thin curves, individual participants; thick curves, average accuracy). Based on the moving average, the trial number required to reach 85% correct performance was calculated for each individual subject. When we compared the average trial number where these smoothed curves reached 85% between the icons associated with a high and low reward, we did not observe a significant difference in the learning rate (paired t-test: t (15) = 0.206, p > 0.25). Thus, although it is evident that the subjects used feedback about the accuracy of their responses because they could not have learned otherwise, the difference between zero and 10 points did not impact strongly on the learning speed.

### Probe task

**Figure 2:**
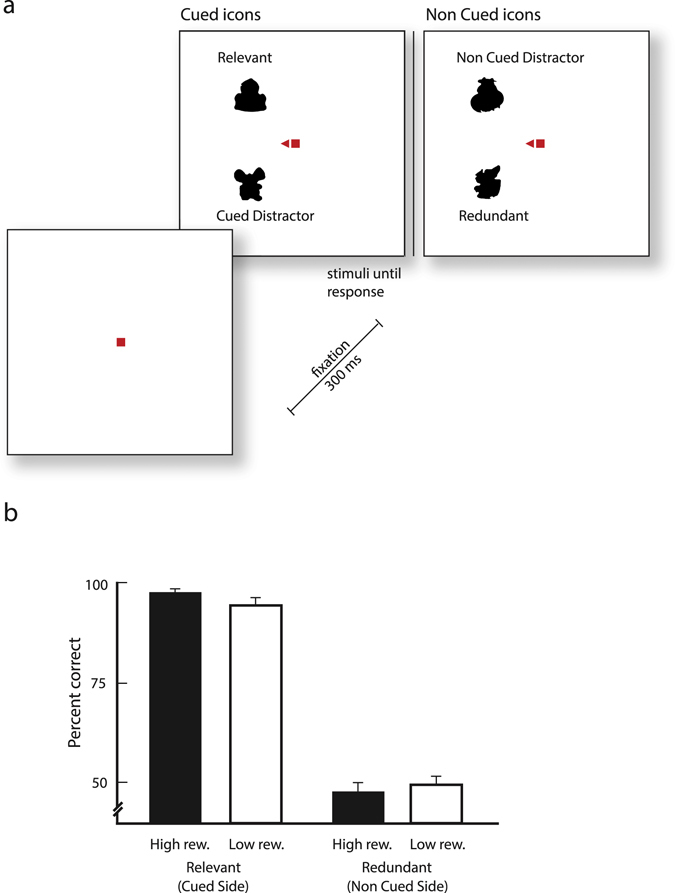
Probe task testing the influence of attention on learning. (**a**) Trial structure. Participants saw icons that had been cued during learning (a relevant icon and a cued distractor) or icons that had not been cued (a redundant icon and a non-cued distractor). We gave no feedback on  whether the response was correct or erroneous to prevent additional learning. (**b**) Accuracy in the probe task for icons that had or had not been cued during learning. Black (white) bars denote the accuracy for icons that had been associated with a high (low) reward. Error bars represent standard error of the mean.

After the shape learning task, participants took part in the probe task which aimed to measure the influence of reward and attention on the associations that had formed during learning. We now presented only the half of the stimulus that the subjects had attended or the half that they had ignored during the learning task. Thus, in trials with the previously attended side, the subjects saw a relevant icon and a cued distractor and in trials with the previously unattended side subjects saw a redundant shape and a non-cued distractor (Fig. 2a). Subjects had to press the button they had learned but they did not receive any feedback about the accuracy of their response, to prevent additional learning. The mean accuracy for the relevant icons was 96 ± 1% (mean ± s.e.m.), whereas the accuracy for the redundant icons was at chance level (48.4 ± 2%) (Fig. 2b). Examination of the effect of reward on the learned association was difficult as accuracy was very close to ceiling for the relevant icons, and the data was highly skewed (i.e. not normally distributed). We did, however, find a subtle effect of previously experienced reward associations using non-parametric Wilcoxon signed rank tests. For the relevant icons, the median accuracy was 98.4% for icons that had been paired with a high reward and 96.9% for icons that had been paired with a low reward, a small but significant difference (Z = 2.12, p = 0.033). There was no significant effect of reward on the accuracy for the redundant icons (Z = 0.85, p = 0.4).

Thus, subjects learned the response mapping only for the relevant (attended) icons, failed to do so for the redundant icons, and the effect of reward magnitude was only expressed for relevant icons. This result is of interest, because the redundant icons were shown equally often, they were equally visible because the subjects maintained gaze at the fixation point, they were equally often followed by the feedback that the response was correct and they were associated with the same amount of reward as the relevant icons. The implication is that attention gated learning, as learning only occurred for the relevant icons.

### Visual search task

**Figure 3:**
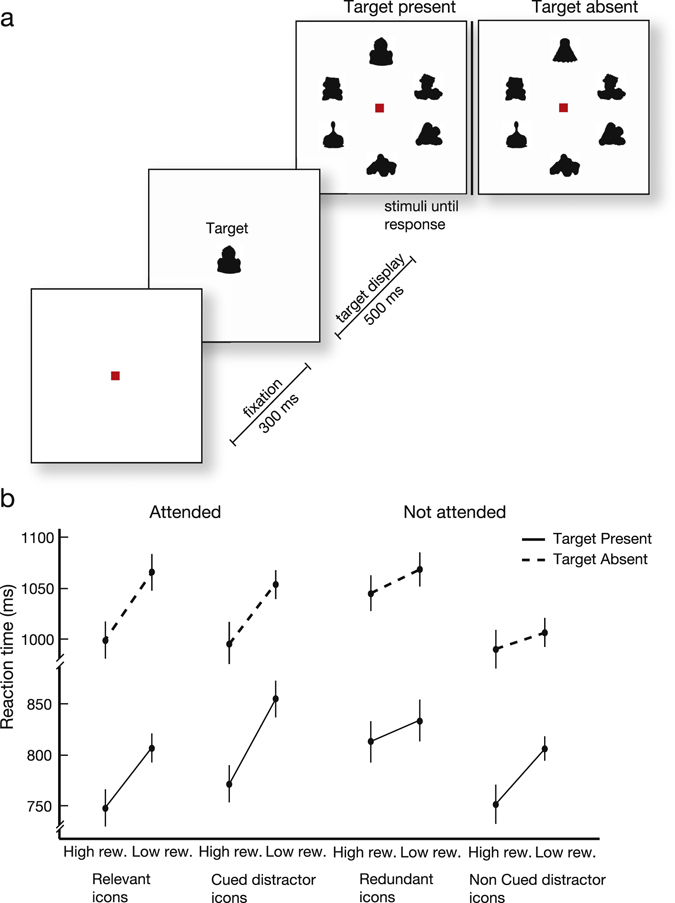
Visual search task. (**a**) Trial structure. Participants indicated the presence of a target icon. (**b**) Mean reaction time on trials in which subjects searched for the previously displayed icons with different reward values. Continuous (dashed) lines, reaction times in target-present (target absent) trials. All error-bars are s.e.m. across subjects.

We did not observe an effect of reward magnitude on the learning speed and it is conceivable that the null effect is related to the stochasticity of the learning process (Fig. 1). Previous studies have demonstrated that an influence of reward can carry over to a subsequent task^17, 18^. We chose a visual search task which provides a sensitive measure for previously established reward associations^17, 19^. The subjects now searched for one of the icons from the learning task. They first saw the search target, which was followed by a search display with six icons that contained the target icon in 50% of the trials (target-present trials) and only distractors in the other 50% of trials (target-absent trials) (Fig. 3a). All the distractor icons were new and had not been used in the original learning task. The subjects performed the search task with an accuracy of 93±1% (mean ± s.e.m.).

We will first describe the reaction times (RTs) in correct trials of the search task (Fig. 3b). We performed a four-way ANOVA with the factors ‘target present/absent’, ‘attention’ (relevant and cued distractor icons vs. redundant and non-cued distractor icons), ‘reward’, and ‘pairing with correct response’ (relevant and redundant icons vs. cued and non-cued distractor icons). As expected in visual search, RTs in target absent trials were longer than those in target present trials (F(1,15) = 58.4, p = 2.0·10^−6^). There also was a significant main effect of the reward level during learning, which confirmed the sensitivity of search tasks for detecting previously established reward associations. RTs for icons that previously yielded 10 points were shorter than those during search for icons that had been worth zero points (F(1,15) = 51, p = 3.0·10^−6^). Although there was no main effect of attention (F(1,15) = 0.02, p > 0.250) there was a significant interaction between attention and reward (F(1,15) = 5.4, p = 0.03), indicating that the effect of reward value was stronger for the attended icons than for the non-attended ones (Fig. 3b). We found no significant main effect for the factor ‘pairing with the correct response’ (F(1,15) = 1.7, p = 0.2), but it interacted with attention (F(1,15) = 12.1, p = 0.003). The other two-way interactions and the three-way interaction were not significant (all ps > 0.25). Although the influence of reward was weaker for the unattended icons than for the attended ones, we asked if there was a residual reward effect for the unattended icons. To this aim, we carried out an additional 3-way ANOVA with only the redundant and non-cued distractor icons. The factors were ‘target present/absent’, ‘reward’ and ‘pairing with correct response’. We observed a significant main effect of reward for the non-attended icons (F(1,15) = 10.8, p = 0.005). Hence, reward magnitude had a weaker, but significant, influence on search speed for the unattended icons as well. We conclude that high rewards during the learning task decreased the reaction times during the subsequent visual search and that attending to the icons during learning amplified this reward effect.

**Figure 4:**
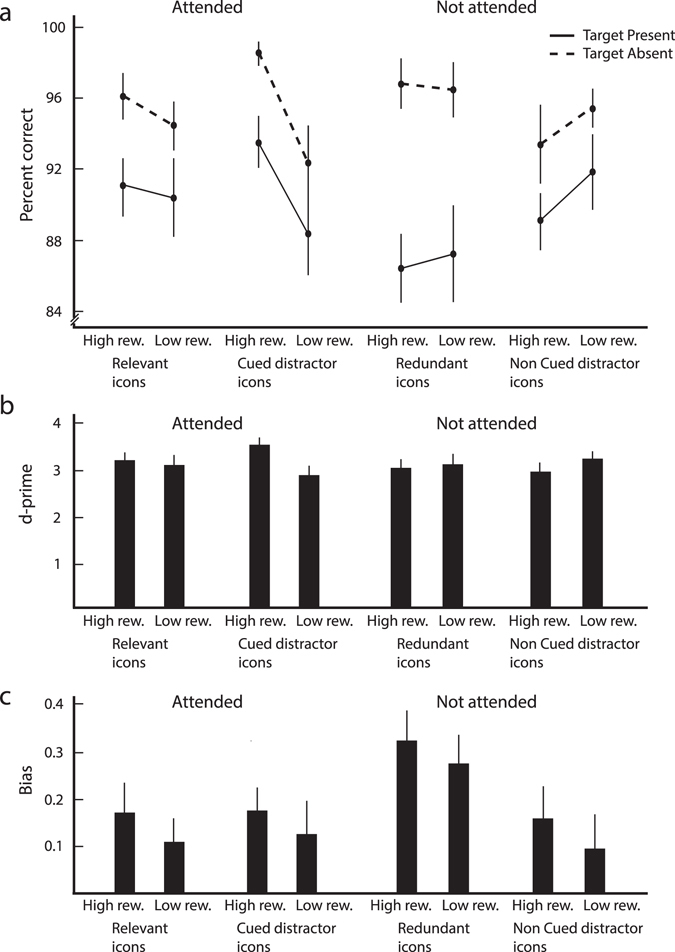
Accuracy in the visual search task. (**a**) Mean accuracy on trials in which subjects searched for the various types of icons of the learning task with different reward values. (**b**) Sensitivity (d-prime) for these icons. (**c**) Bias for the icons. Higher bias values correspond to a more conservative response criterion, i.e. a decreased probability to report “Target present”. Error-bars, s.e.m. across subjects.

An analysis of accuracies with the same factors (Fig. 4a) revealed that subjects were more accurate on target-absent than on target-present trials (F(1,15) = 47.8, p = 5·10^−6^). There was a significant interaction between ‘attention’ and ‘reward’ (F(1,15) = 10.6, p = 0.005) because high rewards increased accuracy for the attended icons. There was also an interaction between ‘target present/absent’ and ‘pairing with the correct response’ (F(1,15) = 7.7, p = 0.014). The other main effects, two-way interactions and the three-way interaction were not significant (all ps > 0.1).

We investigated whether the effects of learning on the subjects’ accuracy was caused by a change in sensitivity (Fig. 4b) or by a change in bias (Fig. 4c) to say “target absent” (Fig. 4c) by applying the signal-detection theory^20^ (as explained in Methods). We performed three-way ANOVAs with the factors, ‘attention’, ‘reward’, and ‘pairing with correct response’ for sensitivity and bias. For sensitivity (Fig. 4b), we observed a significant interaction between ‘attention’ and ‘reward’ (F(1,15) = 11.5, p = 0.004) indicating that high rewards increased sensitivity for the attended icons but decreased sensitivity for the non-attended ones. The other main effects, two-way interactions and the three-way interaction were not significant (all ps > 0.05). An analysis of the bias (Fig. 4c) revealed a significant main effect for ‘pairing with correct response’ (F(1,15) = 6.4, p = 0.023), which was driven by a bias to report ‘target absent’ for the redundant icons. All other main effects, two-way interactions and three-way interactions were not significant (all ps > 0.1).

In summary, we found that RTs were generally faster and accuracies higher for attended shapes that were paired with high reward during the learning phase.

## Discussion

We here developed a new paradigm to test the conjoint influence of reward and attention on the learning of new stimulus-response mappings. We found that the subjects only learned the stimulus-response mapping for icons that were attended and that learning did not occur for non-attended, redundant icons, even though they were equally visible and presented at the same eccentricity. Furthermore, these redundant icons were equally often paired with the correct button response and with the same amount of reward. The present results therefore provide strong evidence for the role of selective attention in the learning of stimulus-response associations. This result is in line with other paradigms demonstrating that selective attention gates learning^6, 8^, a gating effect that was so strong in the present study that learning of a new stimulus-response mapping was completely absent for the unattended icons. Furthermore, high rewards increased the accuracy for the attended icons in the probe task, but the accuracy for the unattended icons was always at chance level, irrespective of the reward level. We found that the effects of attention and reward on learning also carried over to a subsequent visual search task, where responses to previously highly rewarded icons were faster than responses to icons associated with a low reward. High rewards increased the search efficiency for both attended and non-attended icons, but the influence of reward was stronger for the attended icons than for the non-attended ones.

A possibly surprising finding was the absence of a reward effect on the learning rate. The stimulus-response mappings for relevant icons associated with zero points were learned as quickly as those for icons associated with 10 points. This negative finding should not distract from the fact that feedback on whether the button-press was correct or erroneous must have had a strong impact on learning, because the subjects could not have learned without this feedback. This finding therefore suggests that making a correct response was intrinsically rewarding for the subjects, and that it had a much stronger influence on learning than the precise number of points that could be earned. Another factor that may have contributed to the absence of an effect of reward magnitude on the learning rate is that the learning curves of individual subjects had a high variance (Fig. 1b,c). This variance may have masked a possibly weaker influence of reward magnitude on the learning rate. We did not examine the subjects’ strategy for solving the task in detail, but according to our own experience with the task it seems likely that they initially picked one of the two icons on the attended side of a trial at random, and then tested across a number of trials if it was consistently paired with a particular button press^21^. If it was not consistently paired with the required response, the subjects may have picked another icon, and repeated this process until they learned the entire set of stimulus-response mappings. This sampling strategy can explain the high variance of the learning curves and would imply that demonstrating weaker influences on the learning rate would require testing more subjects or more icons per subject. Furthermore, we used a deterministic reward schedule where correct choices for a particular icon were always rewarded with either 0 or 10 points. It is conceivable that probabilistic reward schedules, which tend to prolong the learning process, might amplify the influence of reward magnitude on the learning rate, but this remains to be tested in future work. At the same time, the accuracy for the attended icons associated with a high reward in the ensuing probe task was higher than that for icons associated with a low reward.

We also used a subsequent visual search task because previous studies demonstrated that it provides a sensitive measure for the pairing of stimuli with rewards^17, 19^. Interestingly, the influence of icon learning on reaction times occurred both in target present and target absent trials. Subjects may have created more efficient detectors for the rewarded/attended icons that allowed a more rapid and accurate detection of the target, in accordance with previous studies demonstrating that experience with specific shapes during visual search decreases reaction times for both target present and absent trials^21^. In support of this hypothesis, the sensitivity (d-prime) was increased for the attended icons associated with a high reward, but decreased for unattended icons associated with a high reward. Alternatively, the changes in search efficiency may have been due to higher levels of alertness or arousal on trials in which the target was a previously highly rewarded and attended icon. While we cannot distinguish between these possibilities in the current study, both interpretations are consistent with a learning process that is sensitive to both attention and reward.

Surprisingly, performance was particularly poor for the redundant icons, and subjects showed a bias towards reporting ‘target absent’ when searching for them. The source of this bias is unclear, but it raises the intriguing possibility that subjects suppressed the representation of the redundant icon.

We will now first relate our findings to previous studies on the influence of reward on learning, then we will discuss previous studies on the role of selective attention in learning and we will close by considering the conjoint effect of attention and reward.

### The role of reward in learning

Reinforcement learning theories hold that learning depends on the RPE: the difference between the amount of reward that is obtained and the amount that was expected^1^. If an action is taken that leads to more reward than was expected, the probability of selecting this response in the future should increase. Vice versa, if the outcome of the action is disappointing, the subject should decrease its probability. The finding of neurons in the brain that code for the deviations of reward expectancy and release neuromodulatory substances that influence synaptic plasticity highlighted the importance of reinforcement learning theories for our understanding of the brain mechanisms for learning. One class of neurons that possesses these properties are the dopamine neurons in the ventral tegmental area and substantia nigra. Their activity depends on the RPE^3^ and the release of dopamine indeed influences the plasticity of connections between neurons^22^. Another class of neurons that could fulfill such a role are the cholinergic neurons situated in the basal forebrain, which globally release acetylcholine throughout cortex. A recent study demonstrated that these cholinergic neurons are sensitive to the RPE^4^. Furthermore, the release of acetylcholine enhances the representation of sensory stimuli^23^ and the depletion of cholinergic neurons causes learning deficits^24^.

The influence of rewards on the plasticity of sensory representations in the brain is known to have strong consequences for the performance of human participants. For example, previous studies demonstrated that the pairing of a visual stimulus with a reward can improve the discrimination or detection of this stimulus, while the performance for other stimuli that have not been paired with reward remains the same^10, 22, 25^. Furthermore, visual stimuli associated with a high reward in one task tend to attract more attention in a later task than stimuli that had been associated with no reward^18, 26^. The present results are compatible with these previous findings, because we found that icons that had been associated with a higher reward were learned better and found faster during visual search than the icons associated with a lower reward. Neurophysiological and imaging studies reported a possible neuronal correlate for the enhanced processing efficiency of stimuli previously associated with higher rewards, because these stimuli elicit stronger neuronal activity in areas of the visual^27, 28^ and parietal cortices^29^.

### The influence of selective attention on learning

In our task, selective attention also had a strong impact on the learning of the new stimulus-response mappings. No learning occurred for the redundant icons, even though they were equally often paired with the reward as the attended (relevant) icons. Furthermore, we found that attention amplified the influence of reward on the efficiency in the subsequent probe task and visual search task. Although our focus was on the learning of stimulus-response mappings, our results are in line with studies on the influence of attention on perceptual learning. In a now classical study, Ahissar and Hochstein^6^ trained subjects to perform a task that could be solved by either attending to a specific pop-out line element in an array of line elements but also by attending to the global configuration of the display. They found that perceptual learning only occurred for the attended feature and not for the feature that was ignored. Furthermore, attention gates the changes in tuning of neurons in the visual cortex that occur during perceptual learning^30^. In the present study, we explicitly instructed subjects to attend to one side of the display. However, similar results were obtained with a contextual cueing paradigm^7^, in which subjects carry out a visual search and become faster and more accurate if they have to search through displays that they had seen before. The benefits of seeing the same display again are strongest for the display items with an attended color. Thus, these results, taken together, provide strong evidence that attention to spatial locations or to specific features gates learning, and attention also influences the generalization of learning effects to other features^9^. We note, however, that there are also situations where perceptual learning occurs for stimuli that are too weak to be perceived^10, 31–33^, and we previously suggested that these weak stimuli may escape the control of attention over learning^13^. Here we did not explicitly test how stimulus strength influences icon learning, but it is very unlikely that subjects would have learned the unattended icons had we presented them close to the threshold of perception, e.g. at a low contrast.

In the present study, we focused on the learning of new stimulus-response mappings. Subjects saw icons that were novel but easy to see and to discriminate. We found that attention plays a decisive role in the learning of new stimulus-response mappings, implying that selective attention gates plasticity. This control of attention over learning may help to explain the complex patterns of choices that occurred in a recent study using a weather prediction task with reliable and unreliable cues^21^. Although the investigators did not explicitly cue the subjects to direct their attention, the choices were best explained by assuming that subjects focused on some of the cues and ignored others.

### Attention and reward jointly control learning

Our results demonstrate that selective attention and rewards jointly determine learning. Although many previous reinforcement learning theories proposed that reward should influence learning, the present results provide strong support for theories that suggest that selective attention is an additional factor that gates plasticity^11, 12^.

Previous studies have demonstrated how the learning of stimulus-response mappings changes specific connections in the brain^34^. The present results therefore suggest that rewards and selective attention both influence plasticity. Selective attention appears to enable the plasticity of only some of the connections, as if attentional feedback signals from the response selection stage places “plasticity tags” on those connections that played a role in the stimulus-response mapping. These tags, also known as eligibility traces, could then make them susceptible for the influence of neuromodulatory substances such as dopamine or acetylcholine coding for the RPE, so that only connections that played a role in the stimulus-response mapping are modified^11, 12^. We indeed observed an interaction between reward and attention in the search task, such that the reward effect on learning was amplified by attention, just as predicted by these previous theories. Furthermore, the proposed role of these attentional feedback signals in learning is in line with a study that demonstrated that the effects of feedback connections on visual processing rely on the activation of NMDA receptors^35^, which gate synaptic plasticity.

Learning rules that are based on the conjoint influence of attention and reward are biologically plausible, yet they enable training of non-linear mappings of stimuli onto responses, just like the so-called “error-backpropagation” learning rule that was previously thought to be biologically implausible^11–13^. Error-backpropagation is used nowadays to train deep artificial neural networks with many layers, which recognize object categories in everyday pictures^16^ and perform video and board games at a supra-human level^36^. Recent studies demonstrated that the representations at different hierarchical levels of visual system of humans and non-human primates resemble the representations at equivalent levels of these deep artificial neural networks^37^. It is therefore tempting to speculate that the conjoint gating of learning by attention and reward, as demonstrated here, may also permit the training of the deep networks of the human brain, by enabling learning rules that are equivalent to error-backpropagation.

## Methods

### Participants

We recruited sixteen participants (10 females, 6 males, average age: 23 years, 14 right handed). All had normal or corrected-to-normal vision. We obtained ethical approval from the ethics committee at the University of Amsterdam and all methods were performed in accordance with relevant guidelines and regulations. Informed consent was obtained in writing from the subjects before the experiments. Subjects received a fixed monetary compensation for their time and they could earn an additional monetary reward depending on their task performance at the end of the experiment.

### Stimuli and Apparatus

Stimuli were displayed on a CRT monitor (refresh rate: 85 Hz) at a resolution of 1024 × 768 pixels (screen height = 30 cm and width = 40 cm). We used a chin rest and the subjects were seated at a distance of 58 cm from the monitor. The icons were obtained from the University of Amsterdam Object library^38^ and they were displayed using the COGENT toolbox in MATLAB 2013. The subjects’ responses were registered using a keyboard. We used an Eyelink camera (SR Research, Canada) with a sampling rate of 1000 Hz to monitor eye position.

### Tasks

All subjects performed three tasks in succession and the total duration of the experiment was approximately 2.5 hours. The first task was to learn new stimulus-response associations and it took approximately 80-90 minutes. The second task probed the stimulus-response associations that had formed and lasted approximately 15–20 minutes. The third task tested the influence of learning in a subsequent visual search paradigm and lasted approximately 20–25 minutes.

#### Icon learning task

The subjects fixated on a red square at the center of the screen (0.46**°** × 0.46**°**) and saw four icons in every trial of the learning task (Fig. 1a). The icons were black (0.005 cd/m^2^) and they were displayed on a grey background (15 cd/m^2^). They had an irregular shape and were confined to a square of 5.4**°**. We instructed the subjects to maintain gaze at the fixation point while paying attention to the side of the screen that was cued with a red triangle (0.3**°** × 0.46**°**). A trial was initiated when subjects entered the fixation window (diameter ~2.7**°**) and maintained fixation for 300 ms. Subjects were required to continue fixating until they responded. If the subjects broke fixation, the screen was replaced with a text “Please fixate on the cue” and a key press was required to continue and repeat the trial. In every trial there were four icons, two icons on the cued side and two icons on the non-cued side (Fig. 1a). We instructed the subjects that one of the cued icons was relevant and associated with one of two button responses (‘1’ or ‘2’ on the keyboard) in 100% of the trials, and that the other icon was a distractor that was not consistently associated with a button press. We will refer to this other icon as the “cued distractor” because it appeared on the cued side. Participants also knew that the position of the two icons in the upper or lower quadrant was irrelevant. Their task was to learn by trial and error which icons were associated with a particular button response. Correct responses lead to a reward that depended on the identity of the relevant icon: for some of the relevant icons the subjects could earn 10 points and for other icons zero points could be earned. These points were summed towards a total score. Although the cued distractors were not associated with a particular button response, they were informative about the amount of reward that could be gained because some of the cued distractor icons only appeared on trials where 10 points could be earned and other cued distractor icons only appeared on trials where zero points could be earned.

Two different icons appeared on every trial on the non-attended side of the screen, and they were equally informative as the two icons on the attended side. The redundant icon had the same role as the relevant icon. It was consistently associated with the same response (button ‘1’ or ‘2’) as the relevant icon, and with a reward value (low or high) identical to that of the relevant icon. The non-cued distractor icon had the same role as the cued distractor. This icon was uninformative about the required button response but it consistently appeared only on trials where either 10 or zero points could be earned. Table 1 summarizes the roles of the various icons. We presented a total of 32 icons, with four roles (relevant, redundant, cued distractor and non-cued distractor) and two reward levels. In every trial, we pseudo-randomly selected four of the icons while ensuring that every icon was shown an equal number of times. The associations between the icons, the required responses and the rewards did not vary across the learning session. Furthermore, the subjects knew that every trial had a unique correct response, which was rewarded with either 0 or 10 points.

**Table 1:** Roles and properties of object icons: There were a total of 32 icons, two icons for every entry in the table.

Category	Item	Side	Response	Reward
Relevant	Rel1_High	Cued	1	10 points
	Rel1_Low	Cued	1	0 points
	Rel2_High	Cued	2	10 points
	Rel2_Low	Cued	2	0 points
Cued distractor	cDisA_High	Cued	1/2	10 points
	cDisA_Low	Cued	1/2	0 points
	cDisB_High	Cued	1/2	10 points
	cDisB_Low	Cued	1/2	0 points
Redundant	Red1_High	NonCued	1	10 points
	Red1_Low	NonCued	1	0 points
	Red2_High	NonCued	2	10 points
	Red2_Low	NonCued	2	0 points
Non-cued distractor	nDisA_High	NonCued	1/2	10 points
	nDisA_Low	NonCued	1/2	0 points
	nDisB_High	NonCued	1/2	10 points
	nDisB_Low	NonCued	1/2	0 points

All icons were either associated with 10 (high reward) or 0 points (low reward). Relevant and redundant icons were associated with a particular response (1 or 2) whereas distractors were not (A or B).

Subjects could take as long they desired to respond if they maintained fixation. After every button press, subjects received visual and audio feedback on whether the response was correct or wrong. On correct trials, the subjects could either earn 0 points (low reward level) or 10 points (high reward level). When 0 points were awarded, subjects saw ‘Correct, 0 points’ on the screen and heard the sound of a coin drop. When 10 points were given, subjects saw ‘Correct, 10 points’ and heard a cash register sound. After an incorrect response, they saw ‘Wrong’ and heard a buzzer (see Table 1).

Before the main experiment, the subjects participated in a 5–10 min. training block with icons that differed from the main experimental session. During this training session, they learned to use the attention cue, the logic of mapping a relevant icon onto a response and that the position of the icon in the upper or lower visual field was irrelevant for their task. These training shapes were not used in the main experiment. The training session was followed by the main learning task with eight blocks of 128 trials with breaks between blocks. After every block the attention cue and all icons switched sides. Thus, a particular icon always appeared in the attended hemifield or it always appeared in the non-attended hemifield. Subjects with an accuracy lower than 85% in block 7 and 8 (averaged across 256 trials) performed an additional four blocks. Subjects were informed about their performance in the previous block during the break and were awarded a title depending on their performance (see Table 2). As an added incentive subjects could win up to 15 Euros in addition to the monetary compensation (20 Euros), if they scored higher than all other participants. This additional reward was awarded after all subjects had participated in the experiment.

**Table 2:** Performance based achievement levels: These achievement levels were displayed on the screen during breaks.

Performance in the last 128 trials	Title
Accuracy < 50%	Psychophysics - Apprentice
50% < Accuracy < 70%	Psychophysics - Warrior
70% < Accuracy < 90%	Psychophysics - Champion
Accuracy > 90%	Psychophysics - Wizard

#### Probe task

As both the relevant and redundant icons were displayed during the learning task, it was possible that subjects learned the stimulus-response mapping for both the relevant icons on the attended side and the redundant icons on the non-attended side. The probe task examined the effect of attention on learning. The task had the same eye-fixation instructions and time-structure as the learning task, but we only presented two icons that had been presented on the cued side (relevant icon and cued distractor) or on the non-cued side of the learning task (redundant icon and non-cued distractor) and we did not provide any feedback about the accuracy of the responses. (Fig. 2a). The task consisted of two blocks of 128 trials each and subjects received a break after every 32 trials.

#### Visual search task

The third task examined the influence of reward and attention during the learning phase on the efficiency of visual search. Subjects were required to fixate on a red square (0.46**°** × 0.46**°**) and after 300 ms a target icon was displayed for 500 ms while subjects maintained fixation. This was followed by a search display with six icons. During the search display subjects could freely move their eyes and indicate the presence or absence of the target icon by pressing a button (“1” for target present and “2” for absent). The icons remained on the screen until the subject responded. We chose the target from the set of icons of the learning task, but all distractor icons were novel. Half of the search displays contained the target icon and the other half did not. The experiment consisted of a total of 384 trials and subjects received a break after every 64 trials (Fig. 3a). We examined the reaction times (RTs) in correct trials with ANOVAs.

### Signal detection theory analysis of accuracies in the visual search task

We estimated the subjects’ sensitivity *d′* during the visual search by applying the concepts of the signal detection theory^20^:

1 $${d}^{{\text{'}}}=Z(h)-Z(f)$$

2 $$bias=-0.5\ast (Z(h)+Z(f))$$

where *h* is the proportion of hits, and *f* is the proportion of false alarms. *Z* is the inverse of the cumulative standard Gaussian distribution (with mean 0 and variance 1),

3 $$Z(x)={{\rm{\Phi }}}^{-1}(x),\,{\rm{with}}\,{\rm{\Phi }}({x})={\int }_{-\infty }^{x}\frac{1}{\sqrt{2\pi }}Exp(-0.5{z}^{2})dz$$

Note that according to these equations a more positive bias implies a higher probability to report target absent.

We will make the reaction time and accuracy data available at the Open Science Framework.
